# Supplementary figures and images for: PON-Del predictor for sequence retaining protein deletions
Source: PLoS Comput Biol. 2026 Feb 25;22(2):e1014020. doi: 10.1371/journal.pcbi.1014020 (PMC12959651; doi:10.1371/journal.pcbi.1014020)

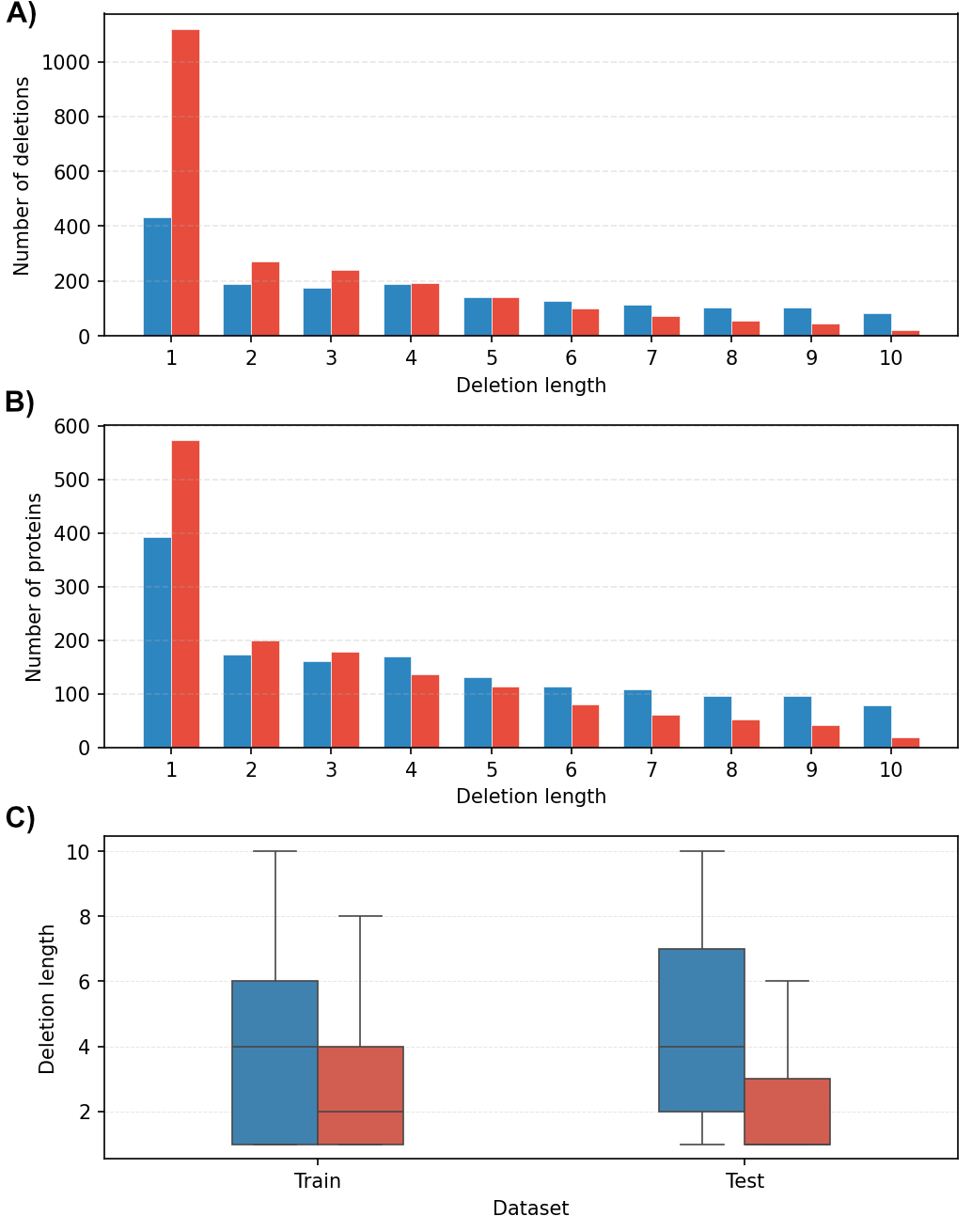

Supplement: S1 Fig — Only variants 10 amino acids or shorter were used for method development due to the low number of longer deletions. Benign variants are in blue; pathogenic variants are in red. A) The deletion length distributions, B) the distribution of the deletion numbers per protein, and C) the length distribution of deletions in training and test datasets. (TIFF) [file pcbi.1014020.s001.tiff]

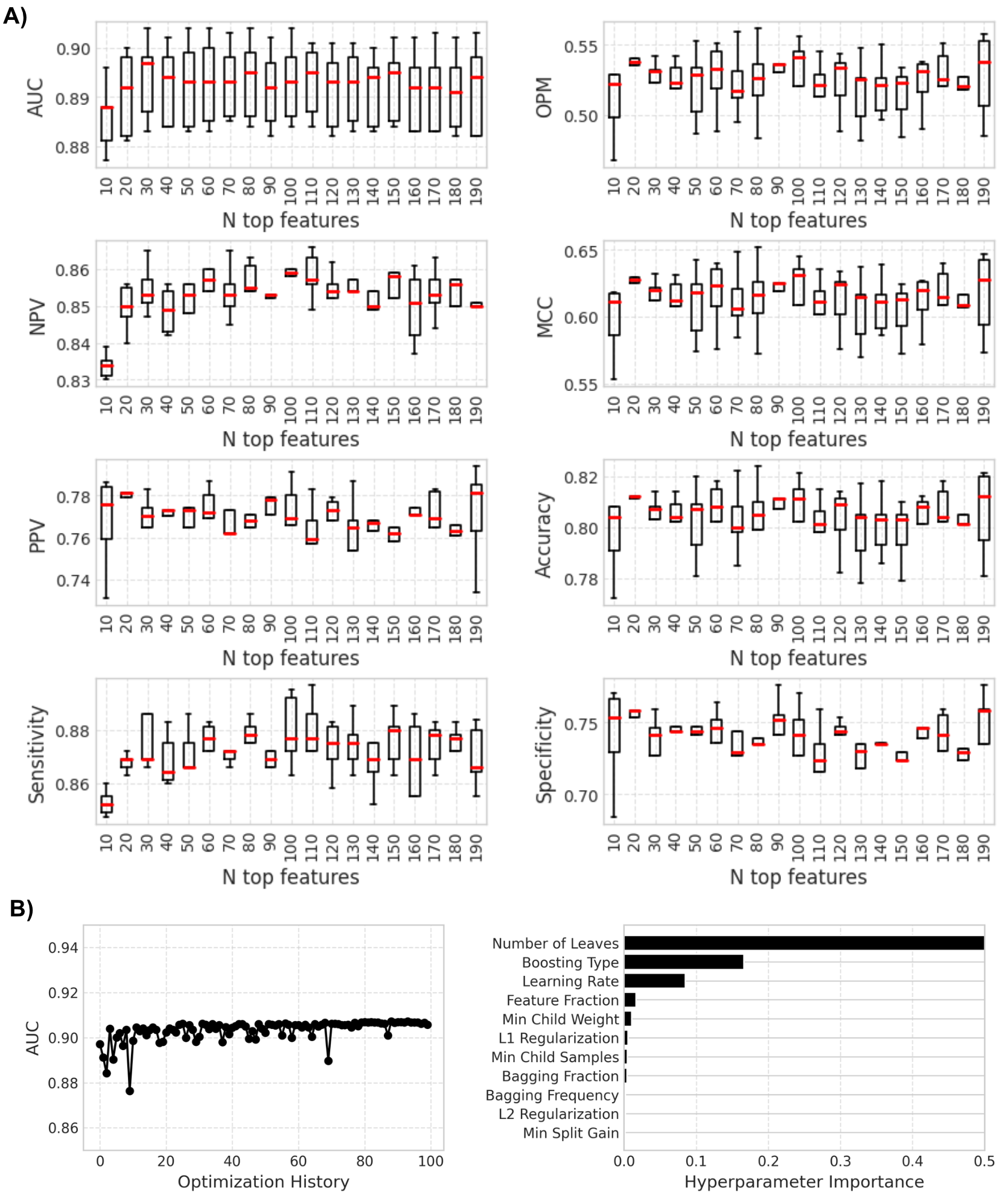

Supplement: S2 Fig — (A) Performance metrics across different numbers of top-ranked features. Red lines indicate the median, and boxplots represent the variability across cross-validation folds. (B) Hyperparameter tuning using Optuna. Left: optimisation history showing the progression of AUC values over 100 trials. Right: relative importance of hyperparameters, indicating that the number of leaves, boosting type, and learning rate contributed most to model performance. (TIFF) [file pcbi.1014020.s002.tiff]

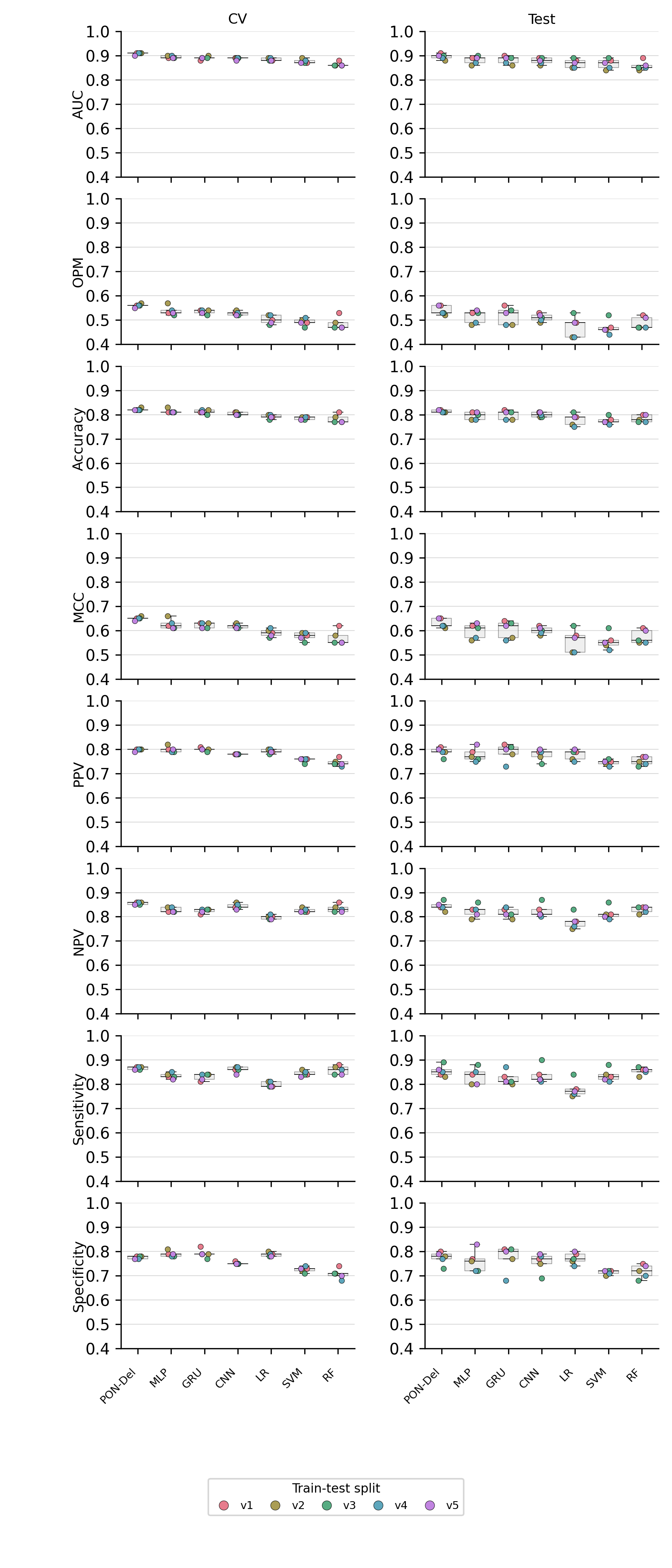

Supplement: S3 Fig — (TIFF) [file pcbi.1014020.s003.tiff]

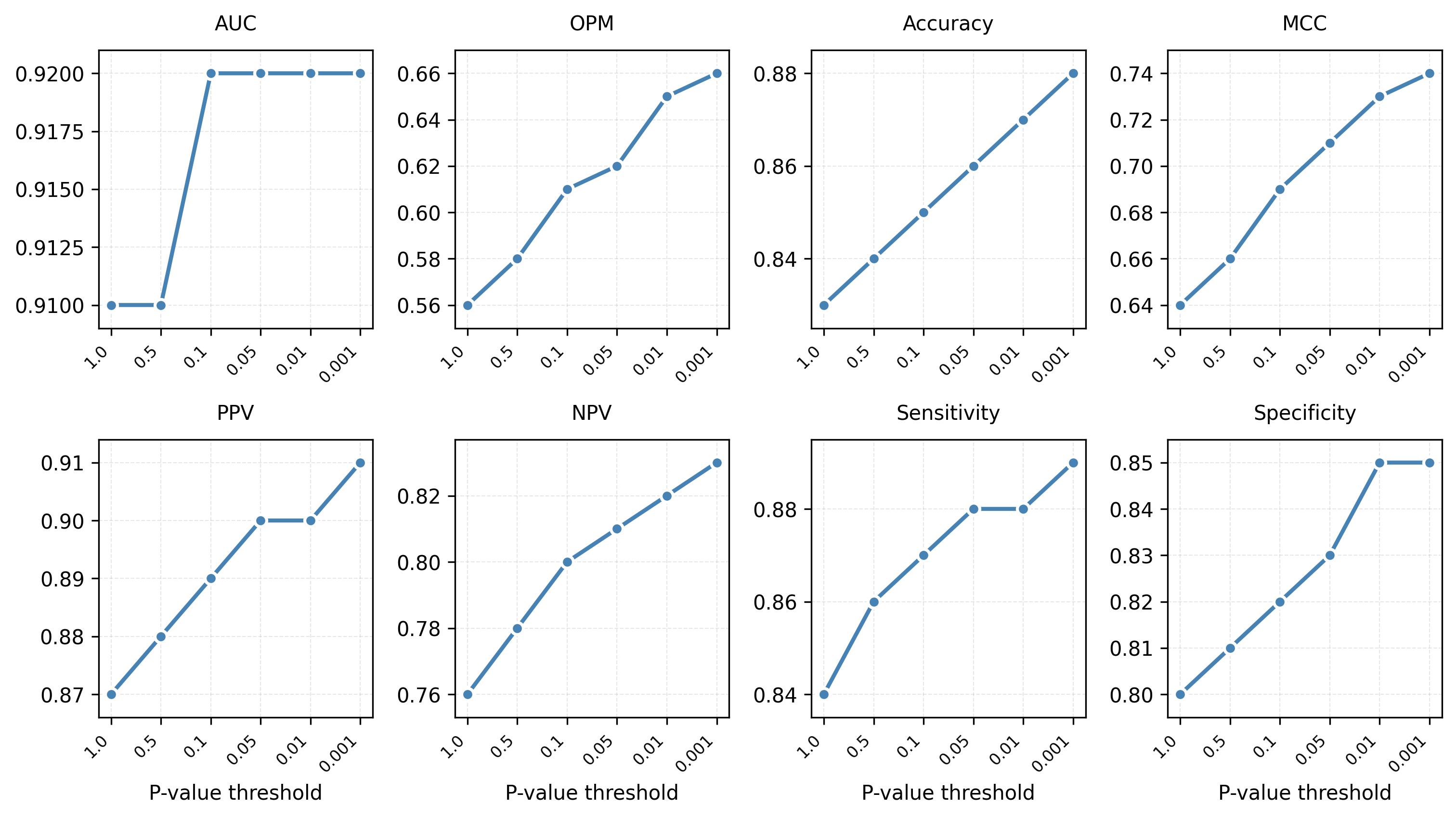

Supplement: S4 Fig — (TIFF) [file pcbi.1014020.s004.tiff]

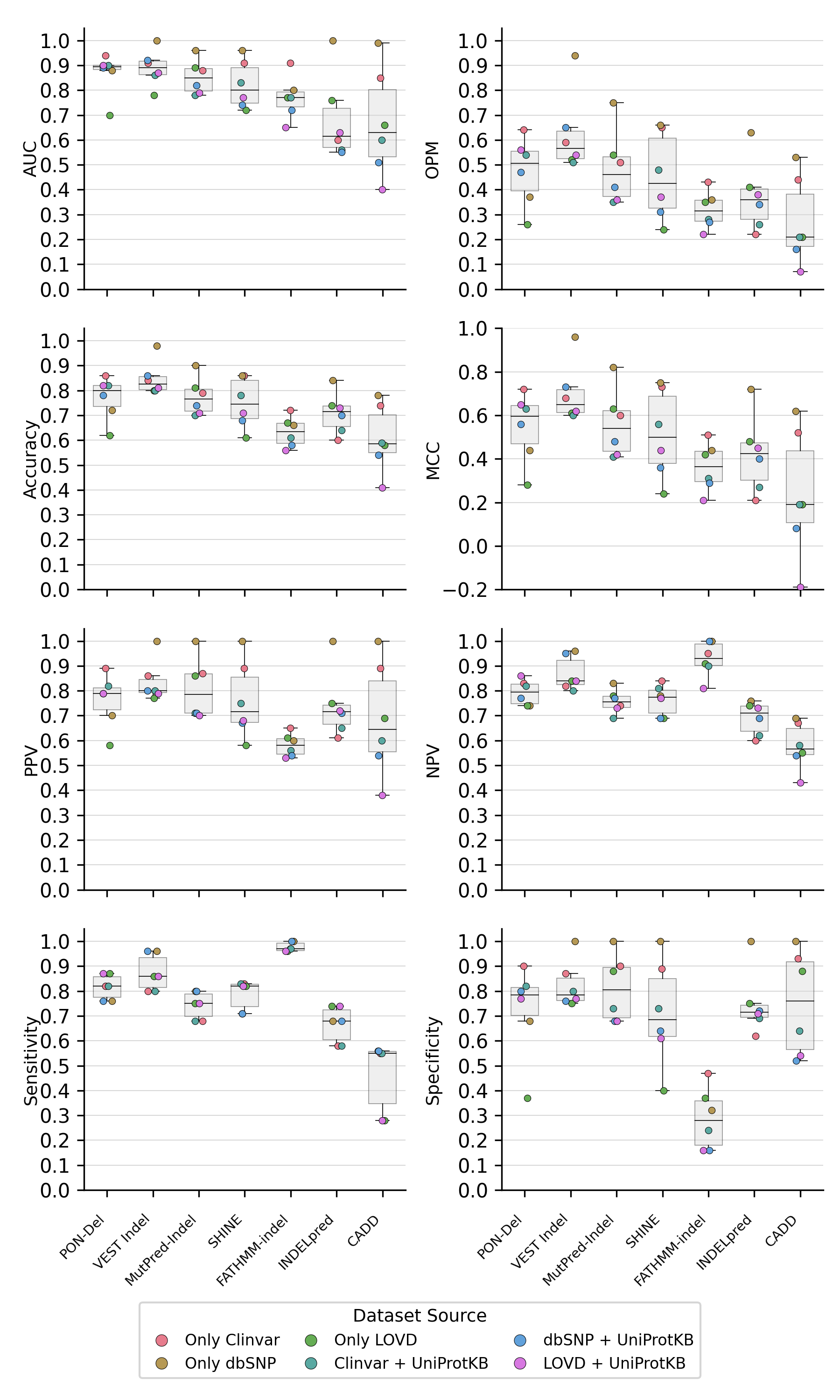

Supplement: S5 Fig — (TIFF) [file pcbi.1014020.s005.tiff]

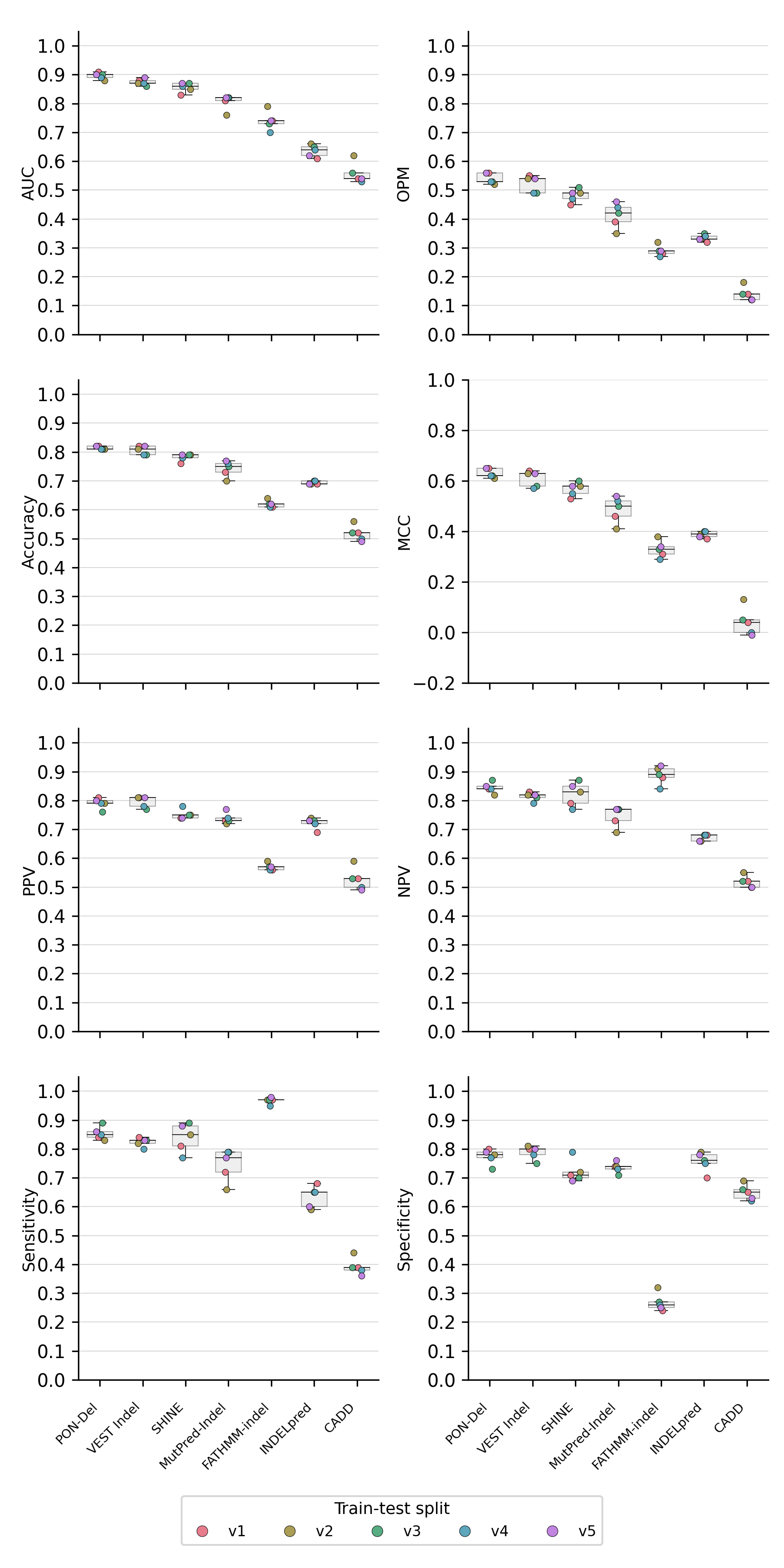

Supplement: S6 Fig — (TIFF) [file pcbi.1014020.s006.tiff]
