# Supplementary material for: PON-Del predictor for sequence retaining protein deletions
Source: PLoS Comput Biol. 2026 Feb 25;22(2):e1014020. doi: 10.1371/journal.pcbi.1014020 (PMC12959651; doi:10.1371/journal.pcbi.1014020)
Supplement: Attachment — Submitted filename: Response letter.docx [file pcbi.1014020.s007.docx]

We are most thankful for the expert review and pertinent comments and questions that have allowed us to improve the text. Our point-by-point replies are as follows.

**Reviewer #1:**

This paper examines diverse machine-learning models and feature sets to build a predictor capable of classifying short sequence-retaining protein deletions as benign, pathogenic or uncertain significance.
1. If the authors aim to answer the question of how a binary classifier can abstain from prediction when it is not confident, threshold-based selective prediction would be an easy and effective approach.

RESPONSE: We fully agree on this note. Our goal was to develop a method with the highest possible performance for clinical and research use, including VUSs. Threshold-based selective prediction is indeed an effective approach.

To assess epistemic uncertainty, we retrained the model using four additional train–test partitions combined with the original split, resulting in twenty-five independently trained models (five folds across five partitions). Then, we applied bootstrap resampling to the probability distributions to quantify prediction stability. The resulting p-values reflect how consistently the model predicts a variant as pathogenic or benign. We measured the performance based on different p-value thresholds, as shown in the Supplementary Figure 4.

Variants filtered out by stricter p-value thresholds tended to be those that are most difficult or unstable, while the remaining variants became progressively easier to classify. Therefore, performance increases monotonically as coverage decreases. This pattern is as expected, because the dataset did not contain experimentally confirmed VUS deletions, since such cases are very scarce. Our three-class output is effectively a selective classifier that abstains when uncertainty is high. For this reason, we adopted a p-value threshold of 0.05.

2. The authors extend a binary classifier into a three-state predictor by introducing a variant of uncertain significance (VUS) category, assigning VUS labels only after the model has been trained. A similar VUS assignment could be added to any competing tool; therefore, the VUS category is not unique to PON-Del.

RESPONSE: Indeed, the third class could be added to any method. However, it has only been applied to PON-Del, making it unique.

3. In Table 4, the performance of the three-state classifier is no better than the binary version on any metric, including false positives and false negatives, which raises questions about the practical value of adding the VUS state.

RESPONSE: The practical value comes from the inclusion of VUSs, since there are three outcomes for variants in relation to diseases. The ability to predict cases into all three categories is beneficial in both research and clinical applications. Information about VUSs can be used, for example, to follow cases that can potentially be reclassified later, and to identify variants that display heterogeneous phenotypes. The latter may require different treatments in different individuals, or no treatment at all for some.

We modified the VUS prediction approach. The original three-state classifier was based on five-fold predictions from a single split and relied on a t-test with a small number of samples, and each fold model was trained on part of the data. The revised predictor uses the full model trained with all training data, and the uncertainty measure is obtained from 1000 bootstrap resampling of twenty-five independently trained models across 5 train–test splits. The obtained estimate is more reliable; however, the predictions are largely the same as with the original approach.

As noted in our response to Comment 1, the three-state classifier operates as a selective classifier. Variants marked as uncertain are precisely those with highly unstable predictions, while the remaining variants are easier to predict and therefore achieve higher accuracy. Table 4 was updated with the new results.

**Reviewer #2**

In this paper, the authors develop a novel software to identify benign and pathogenic mutations from amino acid sequences ranging 1-10 residues. The developed algorithm relied on a deep learning framework trained and tested on a curated database of deletions. The particular framework to generate the prediction and the set of features were selected and optimised to improve the model’s predictive power. This tool was then compared to alternative software, displaying a modest improvement in some performance descriptors but not all. I found that the tool might be useful for the community, but the lack of clarity and the simplistic benchmarking are major issues on the current form of the manuscript.

General comments:
1) The dataset of variants used in this study after filtering consisted of 3912 deletions from 1904 proteins. This was further split into a training set with approx 80% of the data, while the other 20% was used for the blind test. The only specific criteria specified was that no protein was common between training and test sets and that the distribution of deletion lengths were similar. There is no further explanation on how the variants were assigned to each group. Besides, the lack of a cross-validation scheme significantly reduces the reliability of this model. It is not clear, for example, why a 5-fold CV was not used directly in the full dataset. If this was the case, then the proposed tool could be used multiple times to provide a more robust assessment of the tool and the concurrent methods with different training-test pairs.

RESPONSE: We did not apply a 5-fold CV on the full dataset because many proteins share functional annotations, and using all data in the CV could introduce information leakage. A fixed train–test split with protein-level separation ensures generalization to unseen proteins.

We fully agree that multiple train-test splits would be beneficial. We repeated the train–test partitioning four additional times, resulting in 25 trained models. The predictions were combined with bootstrap resampling to obtain the performance estimate. Results are shown in Supplementary Figure 3 and were consistent.

2) While the authors explored which features were learned by the training, a similar evaluation was not performed to understand where the model fails. The outcome of the predictions (TP, TN, FP, FN) was not characterised. It would be important to observe if there are biases on the correctness of the prediction depending on protein families, database of origin, relative position of the mutation, etc. Also, does the (mis)classification pattern is correlated across tools?

RESPONSE: Thank you for the comment. We have previously published an extensive analysis of sequence retaining deletions (PMID: 4121570), where we, e.g., studied functional properties and other characteristics of deletions and proteins containing them. Here, we performed a similar study for correctly predicted and misclassified cases. The results are described in the chapter Interpretability analysis and are supported by Figure 3.

To investigate the impact of the origin of data, we evaluated the classifier separately on variants from ClinVar, dbSNP, LOVD, and UniProtKB. We evaluated the performance on subsets restricted to each source individually, as well as combined with UniProtKB when benign cases were insufficient in numbers (ClinVar + UniProtKB, dbSNP + UniProtKB, LOVD + UniProtKB). The results are shown in Supplementary Figure 5.

3) One of the confusing and maybe misleading information I found is that, in the introduction, there is a detailed differentiation between sequence-retaining and amphigoric deletions. A range of examples on the possible effects of the latter, such as NMD and alternative splicing, were explained. Because the publicly available tool can also analyse transcript and genomic information, it seems that such phenomena can be detected by the software. However, after the introduction, only sequence-retaining deletions are mentioned throughout the text. Overall, it seems that the authors claim that such amphigoric deletions are neglected in most studies, but I do not see how it has been incorporated into this tool, or if it has at all.

RESPONSE: The tool was developed for sequence-retaining variations, as described in the text. There were several reasons. First, sequence retaining and amphigoric variants are entirely different. Second, amphigoric variants are largely misannotated as protein deletions; when in reality, many of them are not produced at all, and therefore cannot be classified as deletions. It would be difficult to obtain a validated dataset. Third, there are not enough cases, especially benign amphigoric variants, for method development. Fourth, true amphigoric deletions are typically harmful and pathogenic, as a substantial part of the protein is missing.

4) The quality metrics (AUC, PPV, NPV, …) were applied to the data and are also displayed in brackets normalised by adjusting the number of benign and pathogenic variants. However, it has been said that most deletions are likely pathogenic. Therefore, this proposed normalisation is far from representing the conditions of a random sample of protein deletions. This can lead to spurious metrics with non-realistic quality assessments. I suggest that this normalisation is either removed or modified to accommodate criteria that better represents the known distribution of benign and pathogenic deletions.

RESPONSE: When reporting the method performances, we followed published and widely followed guidelines for assessment (PMID: 22759650 and 3169447). Indeed, the distribution of most variation types is biased. Amino acid substitutions are mainly benign or VUS, many deletions are harmful, etc. A detailed study has shown that even in such cases, training and testing with a balanced dataset provides the best performance and insight (PMID: 23874456). If results are not normalised, when using an unbalanced dataset, some of the measures are severely affected, see PMID: 22759650.

5) It is not clear what is the impact of the filtering criteria to build the dataset over the assessment across different tools. These choices might negatively influence the performance of concurrent tools. The comparison seems unfair because PON-Del was trained and tested in data following the same filtering criteria, while the other tools did not.

RESPONSE: For this study, we collected a new dataset of deletion variants. The previous datasets may have been less optimal, and affecting the performance of the trained methods. Our performance assessment indicates the true performance of the methods on a validated benchmark. We have included new analyses for performance on the train-test splits in Supplementary Figure 3 and for the effects of deletion data origin in Supplementary Figure 5. The results are consistent.

6) The difference in performance between PON-Del 2-state and PON-Del 3-state is small, but sufficient to make PON-Del 3-state worse than at least one other method in each metric. Therefore, I suggest this differentiation is clear throughout the text and especially in the abstract.

RESPONSE: Thank you for the comment. We have discussed this issue in the Results. Overall, PON-Del 3-state has the best performance, very similar to the 2-state predictor. It is also the most balanced method. Some tools have a single or a couple of good scores, while others are very poor due to overprediction. For example, FATHMM-indel has the best sensitivity and NPV, but specificity is the poorest among the tested methods. The good sensitivity was obtained by severe overprediction.

Once PON-Del is used to classify 3-state patient data, the performance differences to other methods increase further.

Specific comments:

1) Is OPM a standard metric in the field? If so, it should be referenced. Otherwise, it could have been arbitrarily chosen in favor of PON-Del.

RESPONSE: We have added a reference to OPM in Design and Implementation.

2) It would be interesting to see the application to a test case using PON-Del 3-state.

RESPONSE: Thank you. We have replaced Figure 4 with 3-state predictions.

3) In figure 3B, it is not clear how the coloring is done. Maybe use four colors?

RESPONSE: We have added to the legend a key for colors.

4) In table 2, the word metrics has a typo.

RESPONSE: Thank you for the careful reading. It has been corrected.

**Reviewer 3**

The manuscript presents an interesting program addressing a complex challenge in biology: the predicted functional impact of variants on proteins. One of the main strengths of the work is that the authors evaluate several methods on a curated and cleaned dataset, allowing for a fair comparison of prediction algorithms in a specific scenario: namely, the effect of deletions of 1 to 10 amino acids on protein function. However, the main weakness of the study is precisely that this scenario is highly specific included in scenarios of other methods that operate beyond this narrow context and are designed to handle a wider spectrum of variants, including single-nucleotide variants (SNVs), frameshift (inducing deletions and insertions), and other types of indels. In contrast, the proposed method is built around a very constrained situation, i.e. deletions corresponding to amino-acid deletions leading to in-frame amino-acid loss. Interestingly, focusing on this restricted deletion context ultimately shows that some widely used methods already perform as well as possible when constrained to the conditions defined in this study, while still retaining the ability to operate beyond such a narrow context.

RESPONSE: PON-Del is a method dedicated to short sequence retaining deletions. In addition to developing the predictor, we performed an unbiased benchmarking of existing methods by using the validated deletions. Variation interpretation tool developers have different strategies – some develop tools for many types of variations, while others have methods for a single type or effect. We have developed specialized predictors for many types and effects of variations, including human amino acid substitutions (PON-P, PON-P2, PON-P3), amino acid substitutions in any organism (PON-All), effects on protein stability (PON-Tstab), folding (PON-Fold), solubility (PON-Sol), and protein disorder (PON-Diso), amino acid substitutions in mismatch repair proteins (PON-MMR), Bruton tyrosine kinase (PON-BTK), and mitochondrial tRNAs (PON-mt-tRNA). These tools are freely available at https://structure.bmc.lu.se/service/.

General comments on structure and clarity
The structure of the article raises issues of relevance and readability when compared with similar literature.
- The Introduction section contains methodological and concluding elements, which should be moved to the appropriate sections to improve narrative flow.

RESPONSE: We have restructured the text and follow the guidelines for Software articles.

- The Results and Discussion sections are merged, making it difficult to clearly distinguish factual contributions from interpretative aspects. Splitting these sections would make the strengths, weaknesses, and contextual relevance of the method much clearer.

RESPONSE: We have restructured the text and followed the Journal guidelines (which do not contain a separate Discussion section).

Comments on the core method
The proposed method performs at a level comparable to two reference approaches (FATHMM-indel and VEST-Indel). The choice of the PON-Del algorithm appears reasonable, although it does not fundamentally differentiate itself from other prediction methods comparing their validation features (Table 3). This lack of distinction is not explicitly discussed.

RESPONSE: We have added the discussion to the text and addressed this issue. A note on FATHMM-indel. It had excellent NPV and sensitivity, but at the cost of overprediction, visible as the poorest PPV and specificity among the tested tools.

Since all other tools ignore VUSs, in the real-life situation, their performance is lower than the benchmark with two classes indicated. The 3-state test could not be performed due to the limited number of known VUSs among deletions.

Among the available methods, several different algorithmic approaches are represented. Within the gradient boosting category, PON-Del appears to perform better than the other gradient boosting method (aka INDELpred). While other widely used tools such as FATHMM-indel, VEST-Indel, and SHINE achieve similarly strong performance despite relying on different algorithmic frameworks (hidden Markov models, neural networks, and transfer learning, respectively). This should also be presented in a discussion section, apart from the result section.

RESPONSE: It is common in variation interpretation that different algorithms have been applied. Some tools show related performance; some are more divergent. There are many reasons for success/failure, including the choice of the datasets, features used, and feature selection, algorithms used for training, details of training, testing, etc. We comment on this issue in the Results, as this article type does not have the section of Discussion.

On the method validation: all method comparisons rely on a single evaluation using the blind set; however, increasing the size of this blind set and performing a bootstrap analysis across multiple resamplings of the blind set would provide valuable insight into the variability and robustness of performance differences between methods.

RESPONSE: This would be a great improvement, but we already include all the validated cases from the central databases.

Another important point concerns the type of variants used for comparison. The reference methods process nucleotide deletions, which can introduce frameshifts, an aspect explicitly excluded for by PON-Del application. The manuscript rightly focuses on deletions of 1–10 amino acids, but this context-specific applicability must be clearly stated in a dedicated discussion section. The method appears optimal only for this niche use case, and this limitation should be openly discussed, especially since, in routine practice, deletions are not predominantly in-frame. While this does not diminish the technical value of the work, it raises questions regarding its broader applicability.
Finally, the training dataset should be more thoroughly and fairly discussed, including both its strengths and weaknesses. Although the methods section describes how the data were curated, it does not address which genes were included, whether certain genes are overrepresented, the biological processes in which these genes are involved, or the broader biological knowledge that the community has already established about them. This description would strengthen the trust in the training dataset (as well as in the blind set).

RESPONSE: The method was trained for short deletions. The limitation of the deletion length and type is mentioned in several places. There were not enough longer variants for reliable training or benchmarking. Since we could not test and apply PON-Del to longer than 10 amino acid deletions, the usage of the method is limited to this range of deletions.

As mentioned above, Software articles do not have a section for Discussion.

The entire dataset containing details for the included deletions and proteins in which they appear is freely available. The 3912 deletions appeared in 1904 proteins (on average, 2.1 deletions per protein); thus, there is a wide distribution among different types of proteins. The new Figure 4 and added text introduce analysis of the functional and other properties of deletion-containing proteins.

Online tool
The online implementation is user-friendly. However, it does not specify which genome version the tool accepts as input regarding the Genomic Position window. Concerning the output, as noted in the introduction, the handling of protein isoforms is not included. Since the position of an amino-acid deletion depends on the chosen isoform, it is necessary to explain whether and how isoform variability affects the algorithm’s prediction. Or include the multiple algorithm output that refers to isoforms.
The code is available on GitHub (python)

RESPONSE: Thank you for the comment. The method currently works with GRCh38/hg38. We have added notes both to the text and the online tool. As described in the Methods, PON-Del predicts deletions in proteins corresponding to the MANE reference sequences; thus, the deletions must be described in these sequences. This is because some of the features are based on multiple sequence alignments, repeats, palindromes, and 3D structure predictions, which are precalculated (would be too slow to do on the go).

Specific remarks and questions
- “The methods displayed a wide range of performances. The MCC for the best methods was 0.68, far from perfect.” It is unclear whether this is the authors’ own analysis or derived from a referenced study.

RESPONSE: The result is from the published article; the interpretation is ours. We have added a citation.

- Inclusion of VUS is appreciated. However: Can a VUS become pathogenic depending on biological context? How could the algorithm handle this ambiguity?

RESPONSE: VUSs are variants that, according to the definition, either have different phenotypes in different individuals or there is not enough data for classification into other groups. Indeed, a variant can be reclassified. And a variant can be benign in one person while another one with the same genotype has a disease. The VUS prediction aims to identify variants with character patterns that would be related to the heterogeneous phenotype.

- Several sentences in the introduction belong to the Methods, Conclusion, or Discussion sections.

RESPONSE: We have restructured the text to follow the Software article guidelines.

- Figure 1: The different distribution of pathogenic vs. benign variant lengths suggests a potential bias in the original dataset. Or should be discussed.

RESPONSE: The difference is very small and not considered to have any impact.

- Table 3: MetRics (instead of metics); in addition, a bootstrap-based 95% confidence interval would improve the robustness of comparisons.

RESPONSE: Thank you. The typo was corrected.

We agree that incorporating bootstrapping could strengthen the analysis. To address this, we added four train-test splits. We performed bootstrap resampling across 25 trained models. This procedure provides a quantitative measure of uncertainty for each prediction, and the filtered performance metrics at different thresholds are reported in Table 4 and visualized in Supplementary Figures 3 and 6, including the comparison to other tools.

We have also updated our website. All predictions return a bootstrap p-value as a criterion to distinguish VUSs.

- Figure 3B: The red/blue legend is missing. Is the coloring original annotation or the one generated by PON-Del? If the latter, what about VUS classifications?

RESPONSE: The legend has been added for the coloring. Coloring is for predictions and includes VUSs.

- Table 4: Again, confidence intervals (e.g., bootstrapped 95% CI) would strengthen the analysis.

RESPONSE: See our response above to the other comment on bootstrapping.
